# Toward Assay-Aware Bioactivity Model(er)s: Getting a Grip on Biological Context

**DOI:** 10.1021/acs.jcim.5c00603

**Published:** 2025-06-30

**Authors:** Linde Schoenmaker, Enzo G. Sastrokarijo, Laura H. Heitman, Joost B. Beltman, Willem Jespers, Gerard J.P. van Westen

**Affiliations:** † Division of Medicinal Chemistry, Leiden Academic Centre for Drug Research, 4496 Leiden University, Einsteinweg 55, 2333 CC Leiden, The Netherlands; ‡ Oncode Institute, 2333 CC Leiden, The Netherlands; § Division of Cell Systems and Drug Safety, Leiden Academic Centre for Drug Research, 4496 Leiden University, Einsteinweg 55, 2333 CC Leiden, The Netherlands

## Abstract

Protein–ligand interaction prediction with proteochemometric (PCM) models can provide valuable insights during early drug discovery and chemical safety assessment. These models have benefitted from the large amount of data available in bioactivity databases. However, an issue that is often overlooked when using this data is the broad diversity in the biological assays present. The effect of small molecules on a protein can be measured in various ways, and this can influence the outcome. Yet, currently there is a lack of standardized, specific assay metadata, while this could help increase understanding of the origin of data points, improve data curation, and lead to better models that are both more accurate and make predictions specific to the readout of interest. To make use of the existing information on the biological context, we set out to create and validate multiple assay descriptors and test their use in protein–ligand interaction models. Dimensionality reduction of embedded free text assay descriptions from ChEMBL showed that text embeddings capture relevant features. Additionally, clustering of these embedded descriptions groups the assays in a way that enriches purity, matches manually categorized assays, and yields sensible topic describing words. From ligand-protein combinations with multiple measurements, it becomes apparent that the deviation between different measurements in general is higher than the deviation of measurements within assay categories, with a logarithmic mean absolute deviation of 0.83 and 0.66, respectively. Incorporating this biological context into the PCM models in the form of BioBERT-based embeddings improved the average *R* ^2^ from 0.67 to 0.69 across different data sets and splits. Conversely, using simpler methods such as bag-of-words (in which frequently used words are used as features) no improvement was seen (average *R* ^2^ 0.66). Overall, models that integrate assay embeddings yield more accurate predictions and give the user the option to train their model on all available data yet still predict specific end points. In addition, the novel method for assay categorization described here facilitates data curation and provides a useful overview of the biological context of studied targets. In conclusion, biological assay context is important for bioactivity modeling and provides a means to easily get insight into this context.

## Graphical abstract

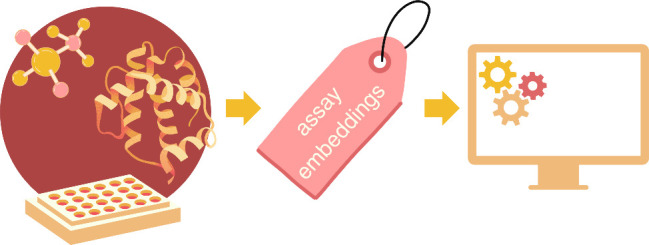

## Introduction

A pivotal step in the drug discovery process is quantifying the interaction of a compound with a protein target of interest. For this purpose, many different biological assays have been developed that quantify these interactions in systems ranging from (radio)­ligand binding assays on cell membranes to functional downstream effects in cellular systems. To capture structure activity relationships in the data gathered by these assays, protein–ligand interaction models have proven to be useful.^1^ These *in silico* methods allow for the identification of compounds with a desired predicted interaction profile and they can increase the efficiency of the drug discovery process. This can be used to enrich for compounds with a high potency on a drug target or with minimal interactions that cause side-effects.

Previous research into protein–ligand interaction prediction has established that grouping data from multiple protein targets improves performance.^2^ These proteochemometric (PCM) models make use of similarities and dissimilarities between proteins in addition to the compound structures used in the target-specific quantitative structure–activity relationship (QSAR) models. This way, the addition of target information increases both model performance and types of applications (for example to extend modeling to several protein targets simultaneously or the inclusion of genetic variants).

An important aspect of creating a data set for modeling is data curation. Bioactivity data sets, such as ChEMBL, make large sets of data from different sources available.^3^ For modeling, it is often assumed that data from different biological assays can be combined. However, this has been criticized and previous research has shown that reported half-maximal response concentrations (IC_50_ or EC_50_) values, inhibitory binding constants (K_i_) and dissociation constants (K_D_) are noisy.^4–6^ Next to experimental error, this could in part be caused by assay heterogeneity; experiments vary in their format, target modifications, detection method, and end point, which can all affect the readout.^7,8^

Recently, multiple studies have been published on ways of combining data from different sources while preserving the assay context. One method to achieve this is by adding generic assay descriptors as an additional model input. The addition of a fingerprint denoting whether the assay is a binding or functional assay, i.e. measures a direct interaction or indirect effects, can lead to improvements in predictive performance.^9^ Another approach that has been explored is multitask modeling, where the model simultaneously makes predictions for various tasks, for example the assay categories, and derives both shared and task-specific parameters. This has been applied on broad assay categorizations such as the distinction between binding and functional experiments,^11^ different readouts,^12^ and on the detailed level of individual publications/records.^9,13^ Taken together, the recent interest in biological assay context has led to an improved predictive performance of models, and has hugely benefitted from the metadata available in ChEMBL. However, for the current metadata in ChEMBL, categorization is either very broad or unique to the specific publication that data originated from. Currently, assay-specific descriptions in ChEMBL are available and provide information on the aim, target and method of the experiment. However, they are present in the form of free text and can therefore not easily be used to group similar assays.

Recent advances in natural language processing (NLP) allow the use of these descriptions for assay categorization. NLP models are well suited for the purpose of processing unstandardized text. The language model BERT, for example, has substantially improved performance within the field of language understanding tasks.^14^ More recently, large language model-based text embeddings have shown state-of-the art performance.^15^ With such pretrained NLP models, meaningful embeddings – numeric vectors representing text – can be created from the assay descriptions annotated in ChEMBL. Learned embeddings based on these descriptions improve predictive quality, in the context of zero- and few-shot learning.^10^ We propose that NLP-based embeddings can also be used in traditional machine learning protocols like QSAR and PCM models. Additionally, with an NLP-based clustering pipeline, BERTopic, assay description-based embeddings can be grouped into topics – with different levels of specificity– allowing for a new way of describing and grouping related biological assays.^16^ Using this form of neural topic modeling, we propose a novel method that leverages biological context in an easily understandable way and show how it can be employed for bioactivity modeling. In cases where the experimental design influences the bioactivity outcome, the inclusion of assay context might improve performance by removing unexplained variance or by predicting the effect at different readout levels.

In this study, we set out to create biological assay descriptions suitable for data curation and modeling purposes. To this end, we examined various methods for incorporating biological assay context, analyzed the difference between biological assays, and evaluated the use of assay context for PCM models on representative sets of target groups. The findings contribute to the incorporation of biological assay context into the bioactivity model and adaptation of this context by the modeler.

## Material & Methods

### Data Set Creation and Preparation

The data and code discussed in this study are available on https://github.com/CDDLeiden/AssayCTX. For clarity an overview of the workflow is presented in Figure 1. To obtain a data set with bioactivity values and annotated assay information, ChEMBL (version 34) was used. Assays were described by the following properties: description, assay_type, assay_tax_id, bao_format, confidence_score, curated_by, pref_name, standard_type.^3^ Only entries from binding (B) and functional (F) assays were kept.^17^ This resulted in 1,277,311 unique assay entries with 1,142,320 unique descriptions. Inspection of the assay description lengths showed that description length is right-skewed and most descriptions have fewer than 500 characters (Figure SI 1). Descriptions beyond this length often contain whole experimental protocols and therefore deviate from most other descriptions. Therefore, we removed assay descriptions when they contained more than 500 characters, a step which filtered out 2441 unique assay descriptions.

**Figure 1:**
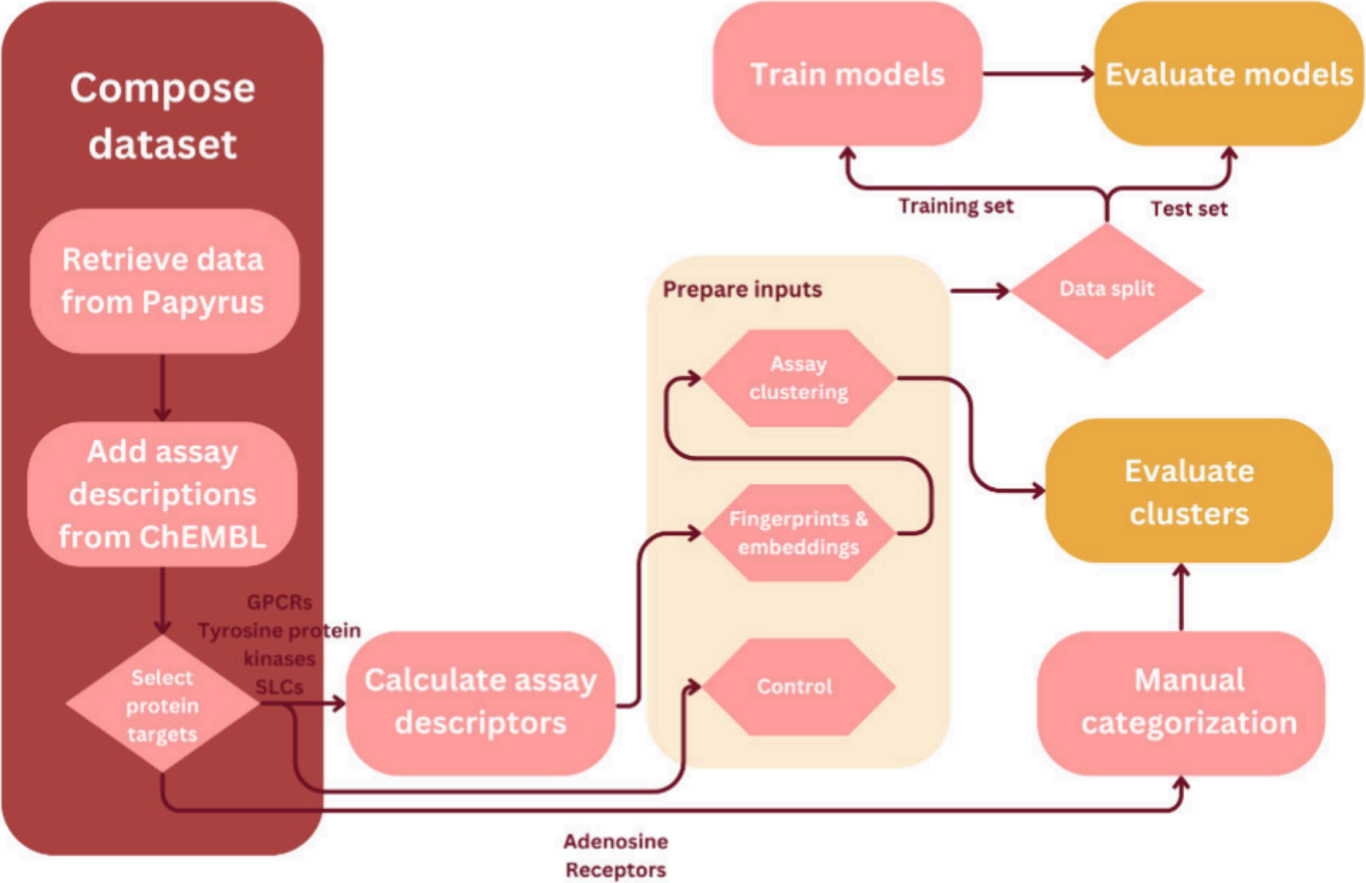
Workflow of the steps taken to create the data set, prepare model inputs, and evaluate the results.

To evaluate the performance of the assay clustering method (described in section “Assay Clustering” below), we annotated biological assays from a small subset of the data. For this purpose, all assays (2476) from the 4 human adenosine receptors (ARs) were collected: AA_1_R (P30542), AA_2A_R (P29274), AA_2B_R (P29275), and AA_3_R (P0DMS8). This subset was deemed small enough for manual annotation and at the same time representative for binding and functional assays recorded in ChEMBL as it includes a variety of different experiments. We labeled the meta target and the detection technology of these assays based on the assay description using subcategorizations previously described in literature.^18,19^ To speed up manual categorization, keywords relating to radioligands, fluorescence methods and functional responses were used for classification. The resulting label frequencies are shown in Table SI 1 and indicate that out of the 13 meta targets the most frequent ones include ligand displacement and cyclic adenosine monophosphate (cAMP) assays, whereas other labels occur less frequently.

To investigate the effect of using biological assay context for bioactivity modeling, we created several representative bioactivity data sets using the Papyrus data set (version 05.7).^20^ Specifically, we collected data for targets from the class A G protein-coupled receptors (GPCRs), protein tyrosine kinases, and solute carriers (SLCs). Only the subset describing ChEMBL data was kept as this can be linked to annotated assay information therein. Low quality data points, i.e. censored bioactivity values and binary activity classes, were removed using the “low quality” data label present in the Papyrus set, as well as compounds with a molecular weight over 1000 Da and data points from publications describing allosteric modulators.^21^ As a final filtering step, only targets with more than 100 unique compounds were kept. As visible from the number of targets, compounds and individual data points, the final data sets of the GPCRs and protein tyrosine kinases are larger than for the SLCs (Table SI 2). The preprocessed data is available on https://zenodo.org/records/15302295.

### Assay Descriptors

Two different types of assay descriptors were evaluated: fingerprints and embedded assay descriptions. Fingerprints were created based on metadata available in ChEMBL and bag-of-words feature extraction. For the fingerprints based on metadata, properties were included if 100% of the property was defined and the property contained 40 or less unique categories (Table SI 3). This was the case for the following properties: relationship type, standard type, src id, confidence score, BioAssay Ontology (BAO) format, curated by, and assay type. For the bag-of-words featurization of the assay descriptions, stemming was applied and English stop words (from scikit learn) and assay specific stop words (see Figure SI 2) were removed. The maximum descriptor length was capped at 1024 bits. On average 7.5 bits (each representing a word) were present per description.

The ChEMBL assay descriptions were also encoded using BioBERT v1.2, a language representation model pretrained on biomedical texts, and gte-Qwen2-1.5B-instruct, a general text embedding model with a high ranking on the Massive Text Embedding Benchmark.^15,22,23^ In both cases assay descriptions of up to 500 characters in length were used. The resulting embeddings consisted of 768 bits and 1536 bits for BioBERT and gte-Qwen2-1.5B-instruct, respectively.

### Assay Clustering

To group similar assays, we performed neural topic modeling based on the assay descriptions available in ChEMBL. To create a generalizable description grouping, clustering with BERTopic was applied to all unique assay descriptions in ChEMBL (with <500 characters).^16^ First, assay embeddings were created using either BioBERT or gte-Qwen2-1.5B-instruct as described previously. These embeddings were then reduced to 5 dimensions using Uniform Manifold Approximation and Projection (UMAP) as is the default setting in BERTopic.^24^ Apart from unsupervised dimensionality reduction, we also tested semisupervised UMAP. To this end, the categorical labels, assay type, BAO format and standard type from ChEMBL were used. The number of neighbors used for manifold approximation was set to 15 and the minimum distance between embedded points to 0.1. These embeddings were then clustered using Hierarchical Density-Based Spatial Clustering of Applications with Noise (HDBSCAN).^25^ In order to vary the level of detail captured by the topics, HDBSCAN was fitted with 16, 32, 68, and 126 as minimum cluster size. Using BERTopic, the word importance within a cluster was assigned with class-based term frequency–inverse document frequency (c-TF-IDF). Descriptions that were not assigned to a cluster after training, hereafter referred to as outliers, were assigned to the most similar cluster based on c-TF-IDF. Because this is a stochastic clustering approach results are given as the mean ± standard deviation of three repeats with different seeds. We have made Jupyter Notebook-based tutorials to show how to obtain these clusters for any ChEMBL data set of interest and how to get information on the underlying pharmacological experiments using a pretrained large-language model (https://github.com/CDDLeiden/AssayCTX/blob/main/assayctx/descriptors/).

### Cluster Evaluation

To evaluate the clustering results, multiple metrics were assessed. First, the clustering results were compared to manually annotated labels for a subset of the data. To quantify the similarity between annotated labels and generated clusters, we used both the homogeneity, i.e., the label agreement of records within the same cluster, and the completeness, i.e., the extent to which all occurrences of a particular experiment are assigned to the same cluster. Second, the similarity of records in the same cluster was assessed based on the normalized purity of the properties assay type, BAO format and standard type. For this purpose the normalized purity was calculated as follows:

$$normalized,purity=\frac{purity-\mathrm{prevalence of the most frequently occurring label}}{1-\mathrm{prevalence of the most frequently occurring label}}$$

This means that the normalized purity of a cluster is one if it only contains objects of the same label, and zero if no label is enriched compared to the whole data set. Third, the weighted average of the mean absolute deviations for protein-compound measurements grouped by assay category was compared to the equivalent deviation inthe whole data set.

### PCM Modeling

To compare the different methods of adding assay context for bioactivity modeling, assay-aware PCM models were trained and evaluated for different protein families. For the control condition, a single task PCM model trained on all agglomerated data without assay context was used. The assay descriptors were evaluated using a single task model with the additional input of the assay descriptor. We evaluated assay-based transfer learning using PCM multitask models, with the tasks corresponding to assay clusters.

For data preparation and modeling, the python package QSPRpred was used.^26^ For all models, the same data sets were used. Bioactivity was expressed in pChEMBL values, i.e., the negative logarithmic of either the scaled half-maximal response concentration, potency, or affinity values. For compound-protein pairs with multiple measurements, either the median pChEMBL value of all measurements was used (control), or the median pChEMBL value of the assays from the same category (multitask) or with the same description (assay descriptor). Molecules were standardized and then described using extended-connectivity fingerprints with a radius of 3 bonds and were folded to 2048 bits (ECFP6). The protein sequences (whole protein, rather than only the binding site) for each protein family were aligned separately via multiple sequence alignment using Clustal Omega (final alignment is available here: https://github.com/CDDLeiden/AssayCTX/tree/main/qspr/data) and described using Hellberg’s z-scales.^27–29^ For the assay descriptor condition, we included additional assay descriptions (as described in the section “Assay Descriptors”). Finally, high correlation (>0.8) and low variance (<0.05) filters were applied to the resulting descriptors from the training set to keep the most informative features. We compared two splitting methods for training set (90%) and test set (10%) creation, based on either a random split or a scaffold split (at the molecule level).

We performed both single and multitask modeling with regression gradient boosting models constructed using pyboost, using the mean squared error (MSE) as the loss function.^30^ For multitask modeling, where the output often consisted of a sparce matrix, missing values were masked. During cross validation, we applied early stopping based on (masked) R-squared (R^2^) values on a holdout set. The models were created using a maximum number of 1000 trees (lower in case early stopping was triggered during cross validation), a learning rate of 0.1, a minimum of 50 data points per leaf, a maximum depth of 8, and a subsample of 80% per tree. Feature importance was recorded based on the gain in RMSE decrease during training.

### Validation

For model validation, the R^2^, root-mean-square error (RMSE) and Kendall’s Tau on the test set are reported as the mean ± standard deviation of three repeats based on different seeds used for the data split. For the multitask models, these values were calculated for each task independently and the weighted average based on the number of data points per task is reported.

## Results

### Natural Language Processed Assay Descriptions Reflect Assay Characteristics

The present study is aimed at incorporating biological assay context into bioactivity modeling. In order to make the assay descriptions that are available in ChEMBL machine interpretable, we compared a bag-of-words and an NLP-based embeddings. In the first method, assay descriptions were converted to a bag-of-words based fingerprint (Figure SI 3). In the second method, the assay descriptions were embedded by the pretrained language models BioBERT and gte-Qwen2–1.5B-instruct. Subsequently, dimensionality reduction was applied to the resulting embeddings. Coloring the data points based on properties from the ChEMBL metadata shows that BioBERT yields informative representations: for several relevant properties a separation is visible among the plotted records (Figure 2) also when the data points are shown as bivariate density estimates (Figure SI 4). The separation is most pronounced for the division between binding and functional assays and for the BAO format. The BAO format shows that within the functional assays there is a clear distinction between the records involving living organisms (orange, organism-based) and cell-based assays (green, cell-based). By coloring on assay tax id (i.e., the assay taxonomy) it also becomes clear that most binding assays and a large part of functional assays are done in human based systems (H. sapiens, red). The majority of experiments done in mice (M. musculus, blue) and rats (R. norvegicus, orange) cluster together and do not overlap with human-based systems. There is a marked overlap between confidence scores and assay types. Interestingly, the different standard types do not separate as clearly as other assay properties, although the readouts minimum inhibitory concentration (MIC), activity, and K_i_ do occur frequently in certain regions. Similarly to the BioBERT-based embeddings, the UMAP of gte-Qwen2–1.5B-instruct embeddings shows local clustering of properties with the same label, although the clusters of the latter are more dispersed (Figure SI 5). Taken together, these results suggest that free text assay descriptions can be transformed into an informative descriptor with NLP-based approaches.

**Figure 2:**
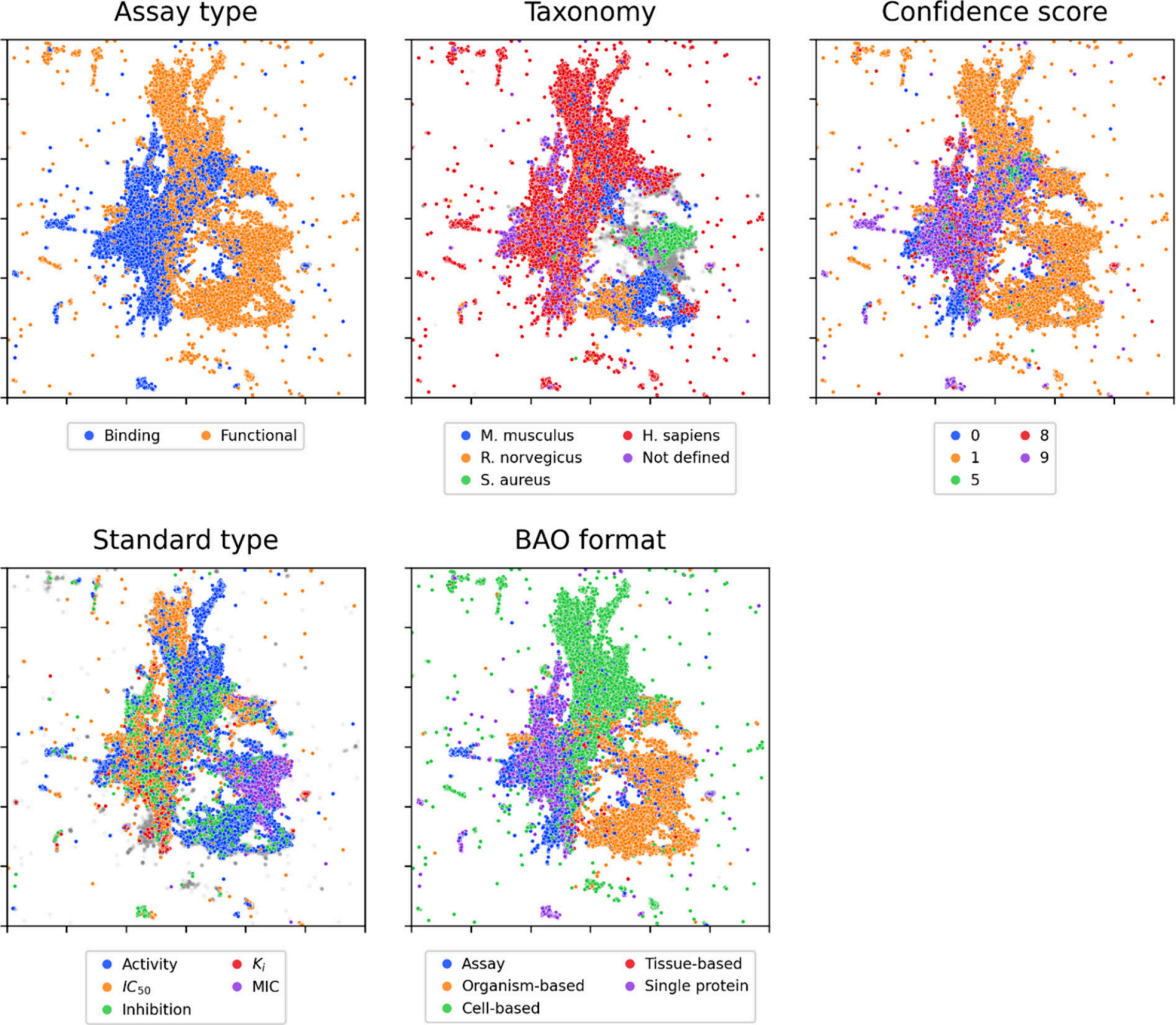
BioBERT embeddings of assay descriptors visualized by UMAP for the categories: assay type, tax id, confidence score, standard type, and BAO format. Data points are colored by the top 5 most prevalent labels; points belonging to other categories are shown in gray. The axes are cropped to exclude highly diverging embeddings.

### Automated Assay Clustering Yields Accurate Groupings

In ChEMBL broad assay categorizations are available but it does not contain groupings of the same – or closely related– assays. Instead, each publication/record is assigned a unique assay ID. To cluster biological assays on the assay level, we created a novel, detailed assay categorization based on embedded assay descriptions. Briefly, we employed UMAP and semisupervised UMAP representations as input for HDBSCAN clustering with a varied minimum cluster size, and c-TF-IDF was used to assign outliers to their closest related cluster.

The clustering performance on the whole data set was evaluated based on the ‘purity’ of the preannotated labels within different categories. Here normalized purity is defined as the sum of the frequencies of the dominant class labels for all clusters divided by the total number of records, and min-max scaled by the prevalence of the most frequently occurring label per property (meaning that no clustering/random clustering leads to a normalized purity of 0). For assay type, BAO format and standard type, the most frequently occurring labels have a prevalence of 0.63, 0.35, and 0.30, respectively. Figure 3 shows that clustering based on BioBERT embeddings increases normalized purity. Records with the same assay type and BAO format are more successfully grouped together than ones with the same the standard type. This analysis also shows that outlier reduction has a beneficial effect on cluster normalized purity and that increasing the minimum cluster size reduces normalized purity. For the gte-Qwen2-1.5B-instruct embeddings the same trends in purity scores are observed but with slightly lower scores (Figure SI 6).

**Figure 3:**
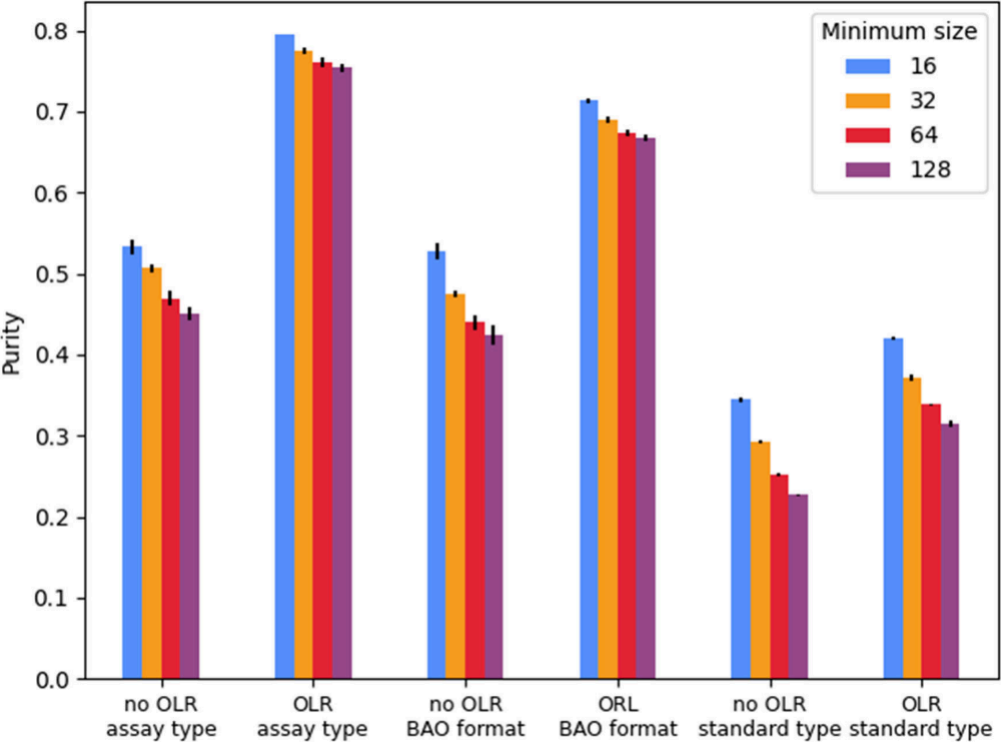
Bar plot of the normalized purity for the labels assay type, BAO format, and standard type based on clustering with BioBERT embeddings. The purity is shown for models with and without outlier reduction (OLR) and with different minimum cluster sizes (16, 32, 64, and 128). Mean and standard deviation are based on independent clustering runs (*n* = 3).

To evaluate the automated clustering method on cluster labels based on manual annotations, we performed manual categorization on records related to the adenosine receptors. The concurrence with this manual categorization was evaluated based on multiple metrics (see Table 1). The best performance in terms of balance between completeness (extent to which members of the same category are assigned to the same cluster) and homogeneity (extent to which a cluster contains members of a single category) was achieved by using BioBERT and a minimum cluster size of 128. Clustering based on gte-Qwen2–1.5B-instruct embeddings results in slightly worse scores. Interestingly, there is a marked difference between the accuracy of clustering for binding and functional assays. The completeness of the functional assays (0.34) is higher than for the binding assays (0.10), which indicates oversegmentation of similar binding assays. For the functional assays a homogeneity of 0.61 is achieved and for binding assays the homogeneity is 0.26. As a side note, the employment of semisupervised UMAP based on assay type or standard type does not improve the clustering accuracy (Table SI 4). The final clustering method (based on BioBERT embeddings and nonsupervised clustering) leads to a total of 1,027 clusters with a median size of 638 records per cluster and the largest cluster containing 24,105 entries. Qualitative evaluation of the topic-describing words that are enriched within the generated clusters (as derived by class-based TF-IDF) shows that they are interpretable and refer to existing assays (Table 2). In summary, these results show that although the clustering is not identical to assigned labels our clustering method gives an accurate and interpretable grouping of the data.

**Table 1:** Clustering Performance Evaluation of Different Minimum Cluster Sizes^a^

	**Type**	**Minimum size**	**AMI** ^b^	**Homogeneity** ^c^	**Completeness** ^d^	**V-measure** ^e^	**Fowlkes-Mallows** ^f^
BioBERT	Functional	16	0.29	0.69	0.25	0.37	0.37
		32	0.29	0.64	0.25	0.36	0.41
		64	0.37	0.64	0.32	0.42	0.53
		128	0.39	0.61	0.34	0.43	0.56
	Binding	16	0.10	0.38	0.10	0.15	0.36
		32	0.09	0.34	0.09	0.14	0.36
		64	0.10	0.28	0.09	0.13	0.41
		128	0.11	0.26	0.10	0.15	0.50
QWEN	Functional	16	0.24	0.60	0.22	0.32	0.33
		32	0.24	0.49	0.23	0.31	0.49
		64	0.35	0.52	0.32	0.40	0.65
		128	0.35	0.45	0.35	0.40	0.63
	Binding	16	0.09	0.38	0.09	0.14	0.29
		32	0.11	0.33	0.10	0.16	0.50
		64	0.09	0.28	0.09	0.13	0.43
		128	0.11	0.23	0.10	0.14	0.59

a Adjusted Mutual Information, measure for agreement of assigned and predicted labels.

b The label agreement of records within the same cluster.

c The extent to which all occurrences of a label are assigned to the same cluster.

d Harmonic mean of homogeneity and completeness.

e The geometric mean of the pairwise precision and recall.

f The performance was evaluated for functional and binding assays separately.

**Table 2:** Top 8 Most Frequently Occurring Clusters Found in the Adenosine Receptor (AR) Dataset^a^

**Cluster ID**	**Total entries**	**Entries in AR subset**	**Topic describing words**	**Predominant assay type (in AR subset)**
1	20199	102	displacement, 3h, from, 125i, membranes, scintillation, counting, dopamine, radioligand, receptor	Binding
3	11550	78	calcium, camp, flipr, forskolin, agonist, intracellular, fluo, mobilization, antagonist, am	Functional
63	2212	770	adenosine, a1, a2a, dpcpx, a3, 21680, cgs, meca, ab, displacement	Binding
192	917	34	cyclase, adenylate, adenylyl, ecd, forskolin, neca, ng108, stimulation, adenosine, moles	Functional
222	1176	185	adenosine, a1, a2a, a3, receptor, a2b, affinity, binding, a2, radioligand	Binding
327	782	355	adenosine, a2a, dpcpx, a1, zm241385, scintillation, counting, a3, displacement, cgs21680	Binding
352	782	283	adenosine, camp, a2b, neca, a2a, a3, a1, cho, forskolin, antagonist	Functional
652	217	88	mre3008, f20, range, a3, adenosine, hek, suppressant, glu, 1st, tyr	Binding

a The total cluster size as well as the number of assays in the subset are shown. For each cluster the topic describing words (based on class-based TF-IDF) are included and the predominant assay type is given.

### Assay Groupings Explain Variance between Measurements

To establish whether inclusion of detailed information on assay context could be helpful for bioactivity prediction modeling, we compared bioactivity outcomes from different assays with overlapping protein-compound measurements (423,195 data points). Plots of pChEMBL values of protein-compound pairs that were measured repeatedly, show a large variability in measured pChEMBL values for the same target-compound combination (Figure 4). The mean absolute deviation for all repeated data points with an associated accession value is 0.83. For GPCRs, tyrosine kinases and SLCs this is 0.76, 1.16 and 0.54, respectively. To establish whether assay context explains part of this variance, the average of the mean absolute deviation within assay clusters derived from the best performing clustering approach was calculated. Overall, this reduces the mean absolute deviation to 0.66 ± 0.02 (based on n = 3 clustering repeats). Notably, the effect of clustering does differ for the chosen target groups. Specifically, the variance of the measured pChEMBL values reduces more for the kinases (mean absolute deviation 0.84 ± 0.09) than for the GPCRs (mean absolute deviation 0.69 ± 0.08) and SLCs (mean absolute deviation 0.47 ± 0.03). In conclusion, biological assay context as extracted with NLP-based clustering partially explains the variance observed in ChEMBL data.

**Figure 4:**
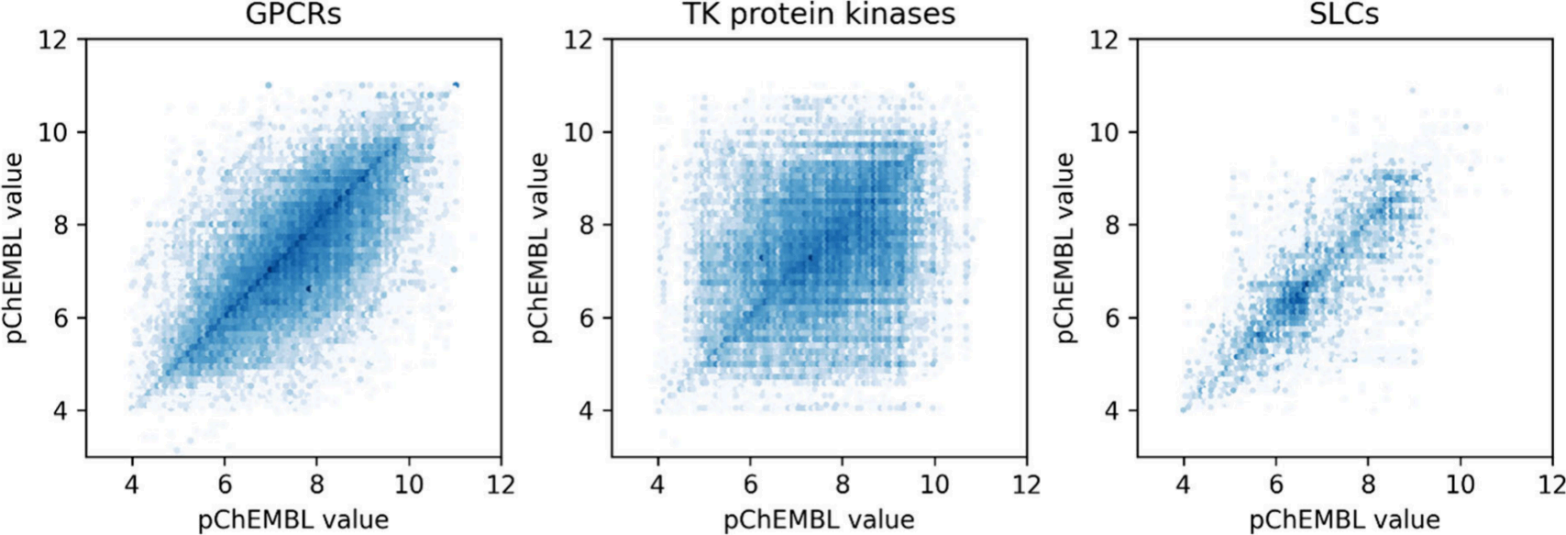
pChEMBL values from compound-protein combinations tested in multiple assays for the GPCRs, tyrosine (TK) protein kinases and SLCs.

### Influence of Assay Context on Modeling Performance

To assess the effect of adding assay context on PCM model performance, we trained and evaluated multiple models. As control condition a single-task PCM model was trained that did not include assay context. This model has an R^2^ of 0.73, 0.68, and 0.69 for SLCs, GPCRs, and tyrosine kinases, respectively. (Table 3, random split). Interestingly, adding a bag-of-words descriptor (a descriptor representing frequently occurring words) consistently slightly decreases model performance, indicating that this might lead to overfitting. The fingerprint based on assay metadata does not affect model performance. For the GPCR case a slight increase in performance occurs when assay description embeddings are used as additional input descriptor (R^2^ of 0.71). This effect is consistent across both the random split and the more challenging scaffold split. Moreover, for GPCRs the RMSE and Kendall’s Tau correlation coefficient exhibit a consistent improvement when using the embeddings. However, no significant improvement occurs upon adding the assay embeddings to the SLC and tyrosine kinase models. Investigation of feature importance shows that assay embeddings are among the most important features for achieving an RMSE decrease during model training (Figure 5). For all targets, the MT models performed worse than the control, indicating that using our assay categories as separate outputs does not improve performance. Overall these results indicate that assay context can improve model performance and that assay embeddings best represent this context.

**Table 3:** Performance of PCM Models with Different Ways of Adding Assay Context Compared to Control^a^

A. SLCs							
		**Random split**			**Scaffold split**		
		R^2^	RMSE	KT	R^2^	RMSE	KT
Control		0.73	0.42 ± 0.02	0.67	0.65	0.58 ± 0.01	0.63
Assay Descriptor	Fingerprint	0.73 ± 0.02	0.41 ± 0.03	0.67 ± 0.01	0.66	0.57 ± 0.01	0.63
	Bag-of-words	0.72 ± 0.01	0.43 ± 0.03	0.6 ± 0.01	0.66	0.57 ± 0.01	0.63
	Embedding	0.74 ± 0.01	0.4 ± 0.02	0.68	0.67	0.56 ± 0.01	0.63
MT		NA	1.10 ± 0.03	0.42 ± 0.01	NA	1.10	0.44 ± 0.01

B. GPCRs							
		**Random split**			**Scaffold split**		
		R^2^	RMSE	KT	R^2^	RMSE	KT
Control		0.68	0.55 ± 0.01	0.63	0.6	0.69	0.58
Assay Descriptor	Fingerprint	0.68	0.55 ± 0.01	0.63	0.62	0.67	0.59
	Bag-of-words	0.65	0.61	0.61	0.58	0.74	0.56
	Embedding	0.71	0.5 ± 0.01	0.65	0.65	0.61	0.61
MT		0.64	0.5 ± 0.01	0.66	0.51	0.64	0.60 ± 0.01

C. Tyrosine kinases							
		**Random split**			**Scaffold split**		
		R^2^	RMSE	KT	R^2^	RMSE	KT
Control		0.69 ± 0.01	0.47 ± 0.02	0.64 ± 0.01	0.68	0.52	0.63
Assay Descriptor	Fingerprint	0.68 ± 0.01	0.51 ± 0.01	0.63 ± 0.01	0.66	0.54	0.62
	Bag-of-words	0.66 ± 0.02	0.53 ± 0.02	0.62 ± 0.01	0.64	0.58 ± 0.01	0.6
	Embedding	0.7 ± 0.02	0.47 ± 0.03	0.65 ± 0.01	0.68	0.5	0.64
MT		0.63 ± 0.03	0.48 ± 0.03	0.64 ± 0.02	NA	0.52	0.63

a Performance is assessed using the correlation (R^2^), root mean squared error (RMSE), and Kendall’s Tau (KT). Mean ± standard deviation of 3 repeats is shown, in cases where the standard deviation is 0 only the mean is shown.

**Figure 5:**
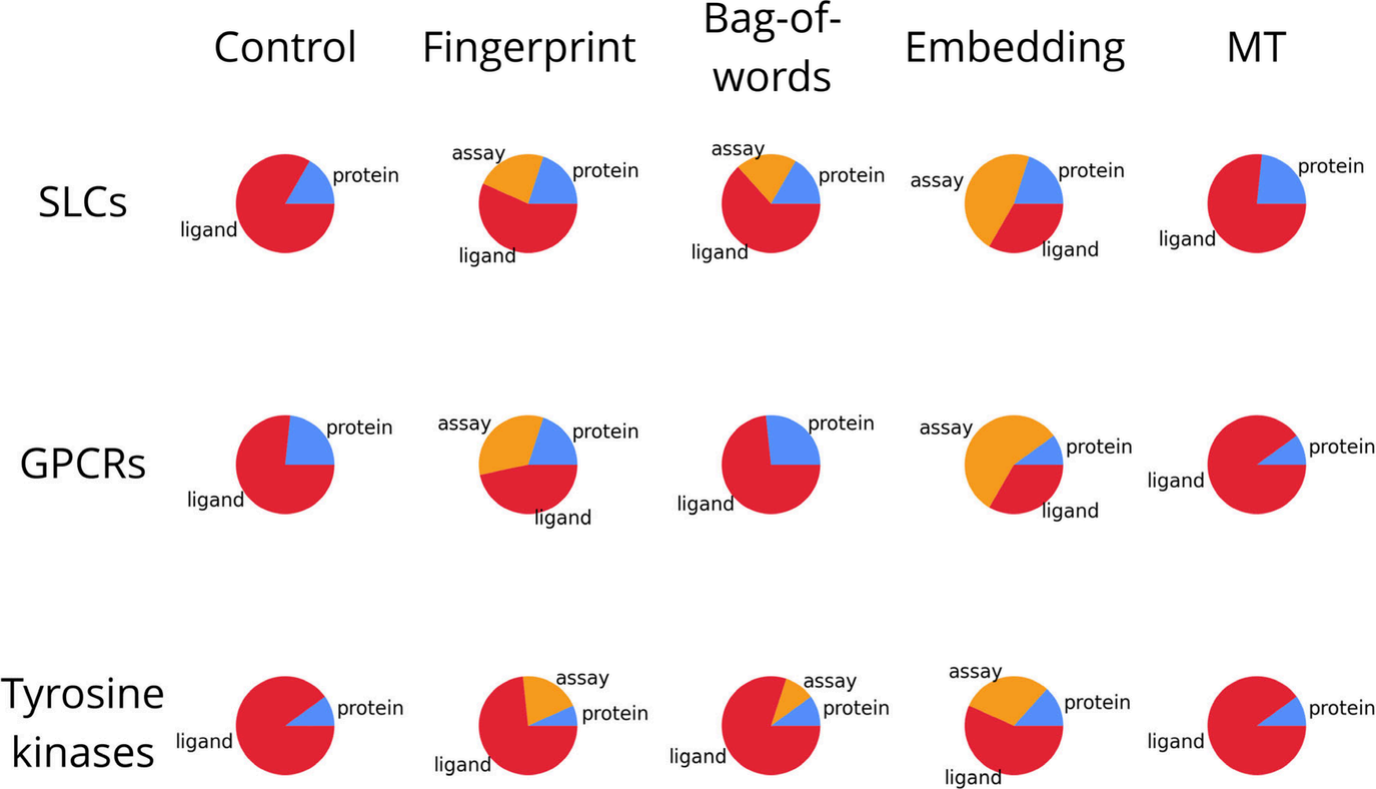
Proportion of different descriptor types for top 30 features based on feature importance. We calculated feature importance by their average gain in mean squared error reduction over 3 repeats for different random splits.

## Discussion

In this research we set out to address the question whether employment of aggregated bioactivity data from different biological assays can be improved by taking this experimental context into account. We demonstrated that one can use NLP techniques to create novel assay descriptors and to give an overview of assays present in a data set. Additionally, clustering of similar biological assays showed that a substantial part of the variance between measurements of the same compound-protein combinations is due to assay context. Finally, including assay context in the form of embeddings slightly improved the predictive performance of PCM models in some data sets.

The main objective of our study was to facilitate the use of the large corpus of written, unstandardized assay descriptions available in the ChEMBL database. To harness this information for incorporating assay context into the bioactivity modeling process, these descriptions were embedded and clustered. For the embedding approach, the pretrained biomedical language representation model BioBERT and the general text embedding model gte-Qwen2–1.5B-instruct were used.^22,23^ The resulting embeddings both capture relevant information as is shown by the concurrence of their mapping with other assay characteristics annotated in ChEMBL. However, BioBERT embeddings show a more concentrated grouping of assays with similar labels. Specifically, a clear division was apparent for the categories assay type, taxonomy, assay format and ChEMBL confidence score. Note that this confidence score reflects the specificity and characterization of the target used for the measurement, hence records with a similar description are indeed expected to have a similar confidence score. The separation based on standard type (i.e., the type of readout) was less strict. This could be caused by the fact that the standard type is not dependent on assay properties like the meta target or detection method but instead on the perturbagen concentration. This information may not be prominently mentioned in the assay descriptions. In general, despite these minor shortcomings these results suggest that the embeddings provide usable information. Further improvements could possibly be achieved with domain-specific large language models like ChemLLM.^31^

In ChEMBL, identical or similar biological assays are currently not grouped together. However, such a grouping could be useful to obtain a clear overview of how the data in a data set was derived, and for modeling purposes. Therefore, one of the objectives of our study was to use neural topic modeling with TF-IDF to cluster similar records, which was successful. It should be mentioned that for all metrics used here we do not expect to see perfect clustering; a perfect way to group – or even label – this very heterogeneous collection of biological experiments does not exist. Overall, the best clustering was obtained for models with a minimum cluster size of 128 (see Table 1) and using outlier reduction (see Figure 3). The normalized purity of labels annotated in ChEMBL showed that the resulting clusters enriched for similar properties, especially when small clusters were allowed. Interestingly, the records that clustered together generally had the same ChEMBL assay type (binding/functional) and BAO format but more diverse standard types. This was in line with the UMAP visualization, in which standard types did not occupy distinct regions. Further evaluation based on comparison to manually annotated labels showed that the assay clusters also grouped similar assays together on a detailed level. Here large minimum cluster sizes perform slightly better than small sizes. In general, the grouping of functional assays performed better than binding assays. One difference between these types of assay is that functional assays are much more diverse than binding assays. The functional assay descriptions, for that reason, often contain highly assay-specific information. Binding assays with different detection methods often only contain detailed information on the radioligand and target which is not relevant for the classification of the assay type. This is for example apparent for the binding assay clusters found for the adenosine receptors, as shown by the receptor and perturbagen specific topic-describing words (Table 2). Overall, our qualitative analysis established that these words made sense and often referred to a specific type of experimental protocol. To facilitate the interpretation of these topics, we have made a Jupyter Notebook that shows how to use a pretrained large-language model to link the keywords to a type of pharmacological experiment (https://github.com/CDDLeiden/AssayCTX/blob/main/assayctx/descriptors/llm_agent.ipynb). It should be noted that the relevance of the assay descriptor is contingent on the specificity of the assay description. Specific assay conditions that can influence the outcome, e.g. the presence of an agonist, can only be taken into account when they are mentioned in the assay description.

This research set out with the hypothesis that different biological assays give significantly deviating outcomes. To evaluate if this hypothesis holds, we established the average deviation between measurements on the same protein-compound combinations. In general, there was a substantial variance between bioactivity values within the same protein-compound pair (mean absolute deviation0.83). This variance was clearly lower when the assay context was considered. Prior studies have also found that results from different assays vary.^5,6,32^ An interesting finding is that a larger variation in outcomes occurred for the kinase data set than for the GPCRs and SLCs. This seemed to mainly be due to assay effects, as the variation within the same assay – as approximated by assay clusters – exhibited a marked decrease especially for the kinase data set. One possible explanation for this finding could be that IC_50_ values reported for kinase inhibitors that are ATP-competitive are affected by the ATP-concentration used in the assay.^33^ Another explanation could be that for kinase inhibitors the binding site of the molecule influences its biological effect, in this work the kinase inhibitor type was not taken into account. It should be noted that we did not look for systematic trends between different assay outcomes. Instead, we focused on using machine learning models to correct for assay specific outcomes.

We showed that including assay context can improve modeling results in some data sets, by explaining part of the variance present in the modeled data. Interestingly, for the kinase data set using assay embeddings on top of the other inputs did not help. Assay descriptions have previously been shown to be useful for modeling by Seidl et al., who found that ChEMBL’s assay descriptions can be used for zero-shot and few-shot learning.^10^ Here we aimed to improve models of targets with sufficient existing data. To this end we utilized multiple PCM models that include assay context. The embedding-based descriptor showed a consistent improvement for the largest data set that was modeled, resulting in an R^2^ increasing from 0.68 to 0.71 in the random split condition and from 0.60 to 0.65 in the scaffold split condition. For most of the other methods and data sets that were analyzed including assay context did not have an effect or even had a negative effect. Specifically, the bag-of-words fingerprint and the models with separate tasks for the assay topics decreased performance. These findings suggest that the heterogeneous nature of assay descriptions requires assay embeddings in order to obtain a clear signal among this noisy information in order to improve performance. For example, to improve the performance for the bag-of-words approach one could attempt to select the used words/bits that improve modeling performance. Alternatively, since the assay descriptions do not always include information that is available in the assay labels (most notably the assay readout), NLP-derived assay embeddings could also be combined with annotated labels to improve performance. The reason that assay embeddings perform well but that MT models with assay topics based on these embeddings have hardly any predictive performance could be due to the balance between data sparsity on one hand and cluster size on the other.

These findings have provided insight into the challenges associated with aggregated data for bioactivity modeling. Currently such agglomerated data is commonly used for modeling purposes and it has been reported that this comes at the cost of a large unexplained variance between measurements.^6^ However, until now, little has been done to address this problem. Here, we demonstrated that measured bioactivity values indeed vary strongly and that part of this variance is explained by the experimental context. Moreover, we showed that the presence of different assays in our data sets negatively influences model performance, although these effects are small and depend on the data set. Prior to our work it was difficult to get an overview of the assays present in ChEMBL and use that information for data curation. With our clustering method, similar assays are grouped and summarized with a few topic describing words. Additionally, we showed that assay embeddings hold information on the assays performed and are therefore valuable to include when modeling data derived from different biological experiments. At inference time, these types of models would be queried with compound, protein and assay combinations to yield predictions for specific end points. A natural follow-up of this work is to apply it in a prospective validation setting.

Being limited to annotated data from ChEMBL and a small subset of manually annotated assays, we could not assess the validity of the majority of found clusters. From the clusters we were able to validate, we concluded that similar assays indeed grouped together, yet also contained some data points where clustering could be further improved. Moreover, in some cases different clusters actually described the same assay, as shown by the completeness score. This was especially true for binding assays, while the resulting readouts are expected to be similar. Part of this oversegmentation could be caused by receptor classes and specific radioligands mentioned in a lot of the assay descriptions. In order to prevent this additional separation based on protein specific information, words referring to such information could be ignored or replaced by a generic abstract term. Ideally, increased awareness of the assay context would help establish validated, standardized ways to deal with this assay context. There is, for example, a definite need for assay-level benchmark data sets.

## Conclusion

Here we have shown that assay context can affect bioactivity modeling outcomes for specific data sets. Our approach enables the use of this context both for the bioactivity model and the modeler. This work does highlight the importance of bioactivity data to be accompanied by well-annotated assay descriptions. Continued efforts should be made to make these descriptions available in a standardized format. Furthermore, follow-up work can likely further improve the handling of free text assay descriptions and therefore of large bioactivity data sets.

## Supplementary Material



## Data Availability

The data and code required to recreate the results of this paper are available in the following GitHub repository: https://github.com/CDDLeiden/AssayCTX.

## Notes

Additional data analysis results including tables on data set composition, (point density) UMAP of BioBERT, bag-of-word and gte-Qwen2–1.5B-instruct embeddings, and additional performance metrics (PDF)
