# Supplementary material for: Toward Assay-Aware Bioactivity Model(er)s: Getting a Grip on Biological Context
Source: J Chem Inf Model. 2025 Jun 30;65(13):7013–23. doi: 10.1021/acs.jcim.5c00603 (PMC12264928; doi:10.1021/acs.jcim.5c00603)
Supplement: Supplementary file 1 [file ci5c00603_si_001.pdf]

Toward Assay-Aware Bioactivity Model(er)s: Getting a Grip on Biological Context

Linde Schoenmaker[1], Enzo G. Sastrokarijo[1], Laura H. Heitman[1, 2], Joost B. Beltman[3], Willem Jespers[1], and Gerard J.P. van Westen[1].*

1. Division of Medicinal Chemistry, Leiden Academic Centre for Drug Research, Leiden University, Einsteinweg 55, 2333 CC Leiden, The Netherlands
2. Oncode Institute, Leiden, The Netherlands
3. Division of Cell Systems and Drug Safety, Leiden Academic Centre for Drug Research, Leiden University, Einsteinweg 55, 2333 CC Leiden, The Netherlands

* Email: gerard@lacdr.leidenuniv.nl

Supporting information

Adenosine receptors assay categorization

Table SI 1, number of documents for different meta target and detection technology categories for the four human adenosine receptors.

**Meta target**	**counts**
ligand displacement	1880
cAMP	383
not specified	121
GTPPgammaS	34
Ca2+	32
ERK1/2	9
inflammation	5
IP	4
oxidative stress	2
morphology	2
beta-arrestin	2
beta-galactosidase	1
cell death	1

Composition modeling datasets (per target group)

Table SI 2, number of targets, compounds, and datapoints for the three final receptor sets.

	**# targets**	**# compounds**	**# datapoints**
SLCs	31	15746	29437
Protein tyrosine kinases	59	49801	95750
GPCRs	247	140220	261903

Number unique and fractions defined of all ChEMBL assay properties

Table SI 3, characteristics of annotated assay properties. The number of unique categories per property is given as well as the percentage of values that is defined.

	**Number unique**	**Fraction defined**
chembl_id	1277311	1
relationship_type	6	1
standard_type	5	1
pref_name	11735	1
aidx	465500	1
src_id	40	1
description	1142320	1
confidence_score	10	1
bao_format	14	1
curated_by	3	1
desc_length	1352	1
assay_tax_id	3683	1
assay_type	2	1
year	49	1.00
doi	85145	0.98
journal	211	0.97
pubmed_id	79843	0.93
assay_organism	4041	0.92
assay_cell_type	6187	0.38
cell_id	1988	0.33
src_assay_id	329676	0.27
assay_strain	25581	0.15
assay_tissue	465	0.06

tissue_id	435	0.06
assay_test_type	3	0.03
variant_id	2398	0.01
assay_subcellular_fraction	86	0.01
assay_category	13	0.00

Adenosine receptor assay categorization performances

Table SI 4, clustering performance metrics of semi-supervised clustering methods based on BioBERT embeddings.

**Type**	**Minimum size**	**Semi-supervised**	**AMI[1]**	**Homogeneity[2]**	**Completeness[3]**	**V-measure[4]**	**Fowlkes-Mallows[5]**
F	128	No	0.39	0.61	0.34	0.43	0.56
F	128	Assay type	0.40	0.62	0.35	0.45	0.61
F	128	Standard type	0.37	0.56	0.34	0.42	0.62
B	128	No	0.11	0.26	0.10	0.15	0.50
B	128	Assay type	0.11	0.25	0.10	0.14	0.52
B	128	Standard type	0.10	0.25	0.09	0.14	0.50

The performance was evaluated for functional and binding assays separately.

1. Adjusted Mutual Information, measure for agreement of assigned and predicted labels
2. The label agreement of records within the same cluster
3. The extent to which all occurrences of a label are assigned to the same cluster
4. Harmonic mean of homogeneity and completeness
5. The geometric mean of the pairwise precision and recall

Example assay descriptions for adenosine receptors

Table SI 5, example of ChEMBL assay descriptions we have categorized as being from cAMP assays

DOI	Description
10.1016/s0960-894x(03)00090-8	Effective concentration against human MT2 (Melatonin) receptor stably expressed in NIH3T3 cells in adenylyl cyclase assay
10.1016/j.bmcl.2004.08.035	Effective concentration required against human histamine H3 receptor was determined by the inhibition of the cAMP stimulated beta-galactosidase transcription in SK-N-MC cells
10.1016/j.ejmech.2021.113907	Antagonist activity at human adenosine A3A receptor expressed in CHO cells assessed as inhibition of cAMP accumulation
10.1016/j.bmcl.2014.11.003	Agonist activity at human CB1 receptor expressed in CHO-K1 cells assessed as inhibition of forskolin-stimulated cAMP production

Frequency plot assay description length

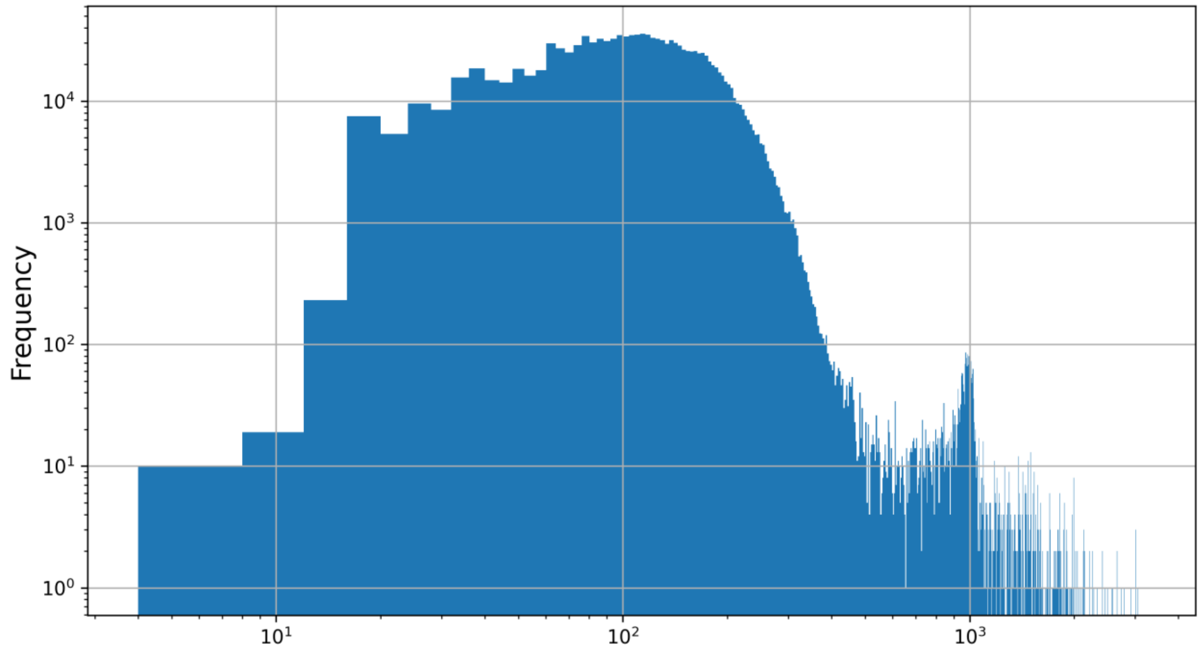

Figure SI 1, frequency plot of assay description lengths based on all unique assay descriptions.

Assay stop words

```
  assay_stop_words = set(['treated', 'kg', 'reduction', 'model',
'production', 'day', 'residues', 'substrate', 'method', 'on', 'level',
'challenge', 'induction', 'type', 'decrease', 'infection', 'for',
'tested', 'with', 'increase', 'presence', 'dilution', 'in', 'evaluated',
'from', 'as', 'assessed', 'dosed', 'ug', 'prior', 'before', 'ml', 'nm',
'inhibitory', 'determined', 'mm', 'control', 'days', 'dose', 'residual',
'based', 'expressed', 'expressing', 'activation', 'origin', 'using',
'length', 'expression', 'concentration', 'compound', 'of', 'incubated',
'up', 'percent', 'formation', 'measuring', 'index', 'post', 'the',
'analysis', 'mins', 'mg', 'hr', 'effect', 'phase', 'test', 'time',
'mic', 'activity', 'min', 'harboring', 'at', 'stimulated',
'administration', 'addition', 'and', 'administered', 'system',
'followed', 'to', 'assay', 'measured', 'mediated', 'by', 'against',
'after', 'um', 'induced', 'hrs', 'stimulation', 'was', 'change'])
```

Figure SI 2, list of assay specific stop words.

UMAP bag-of-word embeddings

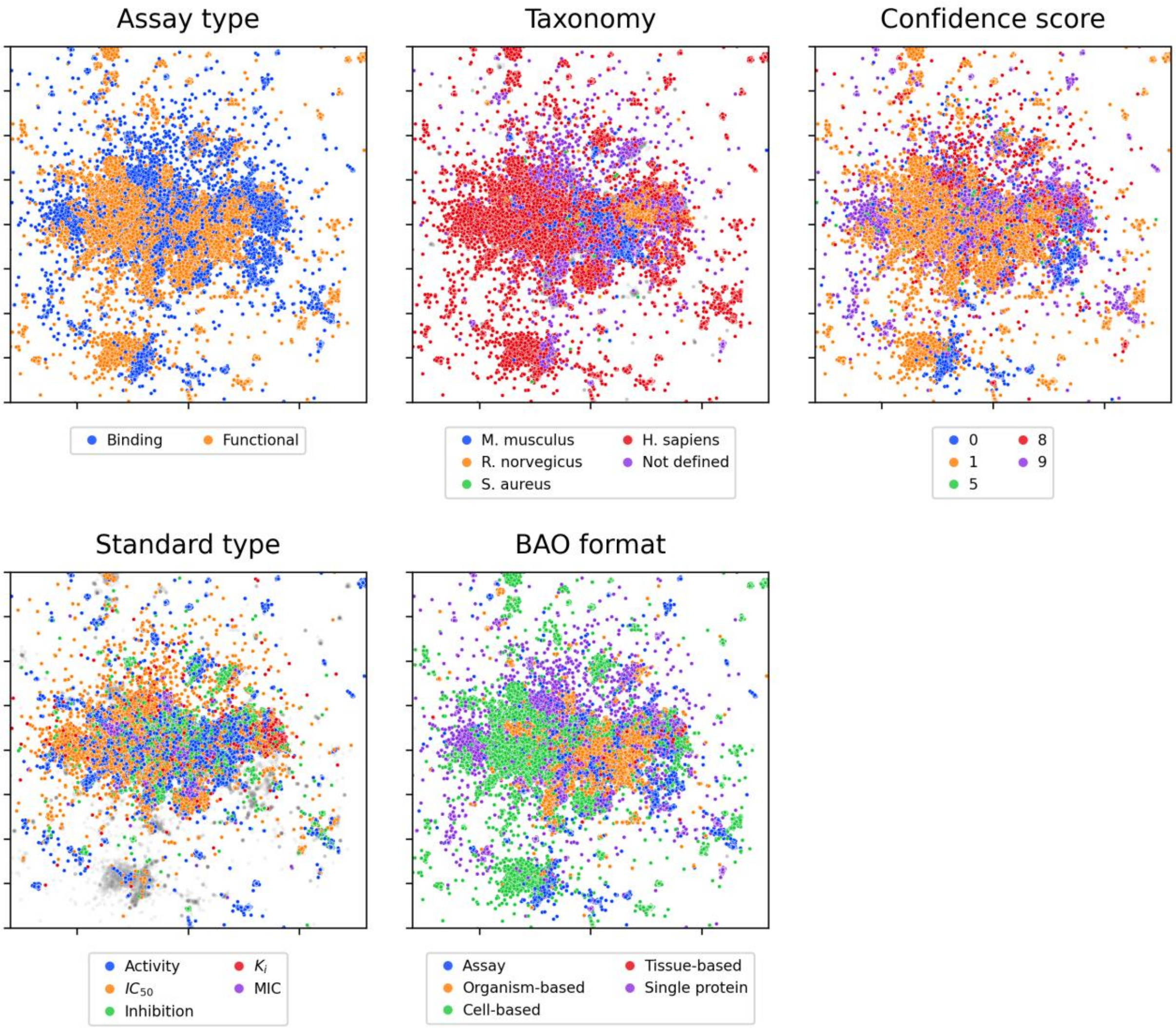

Figure SI 3, UMAP of bag-of-words embeddings. Cropped to exclude highly diverging embeddings.

Density estimation UMAP BioBERT embeddings

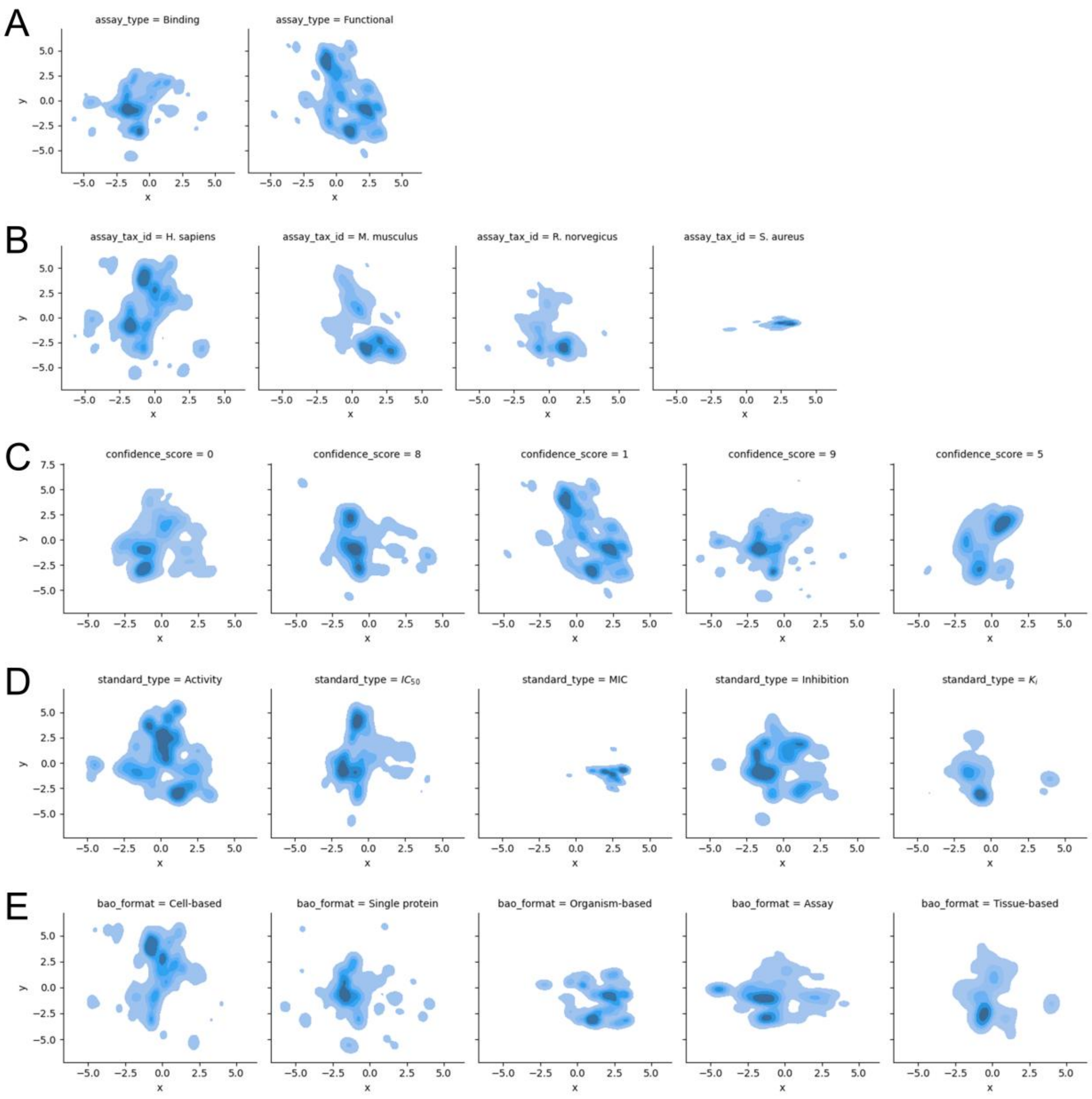

Figure SI 4, point density UMAP of BioBERT embeddings. Density estimates based on kernel density estimation are as shown as contour lines. Cropped to exclude highly diverging embeddings. A: assay type; B: taxonomy; C: confidence score; D: standard type; E: BAO format.

UMAP gte-Qwen2-1.5B-instruct embeddings

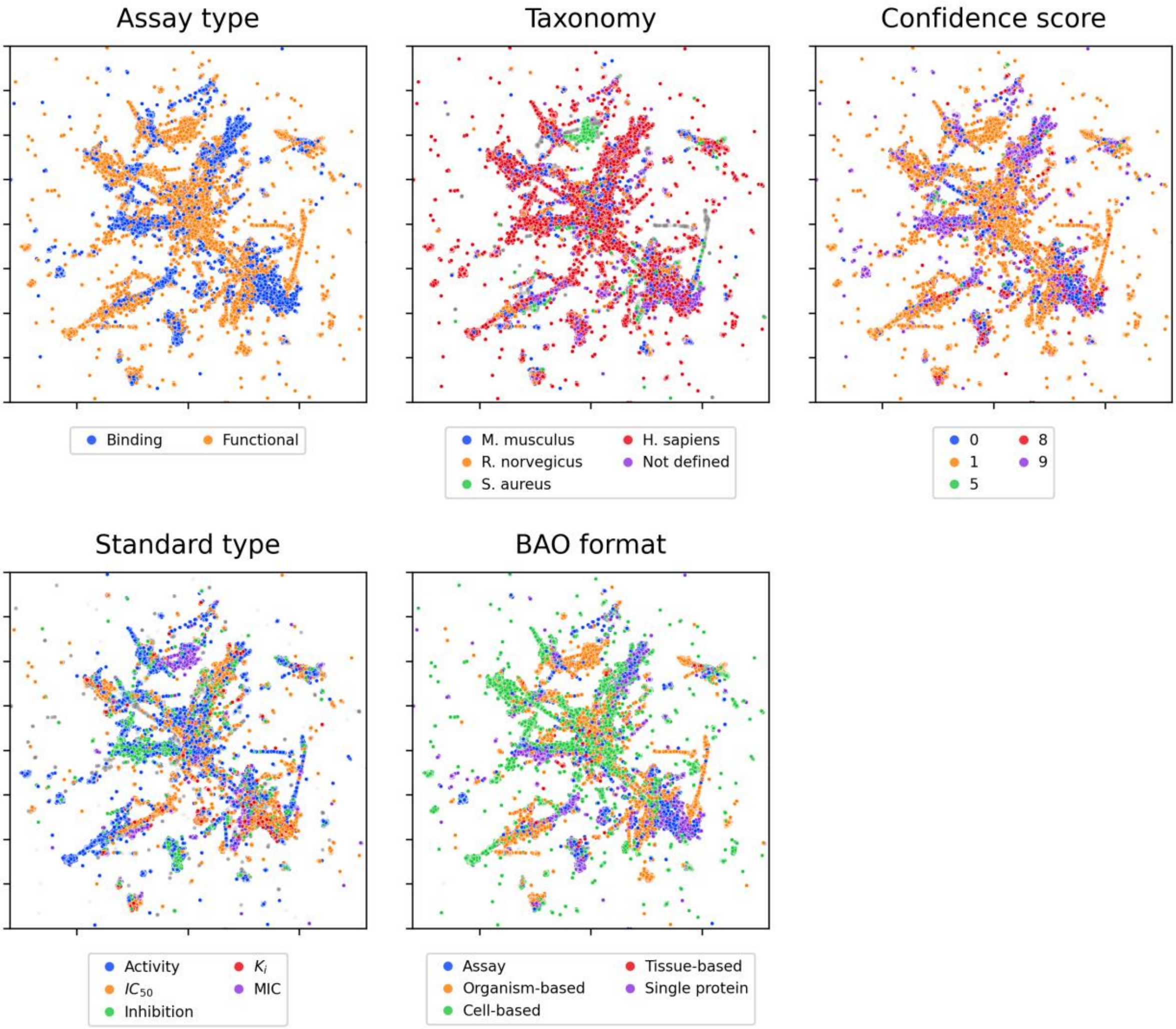

Figure SI 5, UMAP of gte-Qwen2-1.5B-instruct embeddings. Cropped to exclude highly diverging embeddings.

Purity based on clustering with gte-Qwen2-1.5B-instruct embeddings

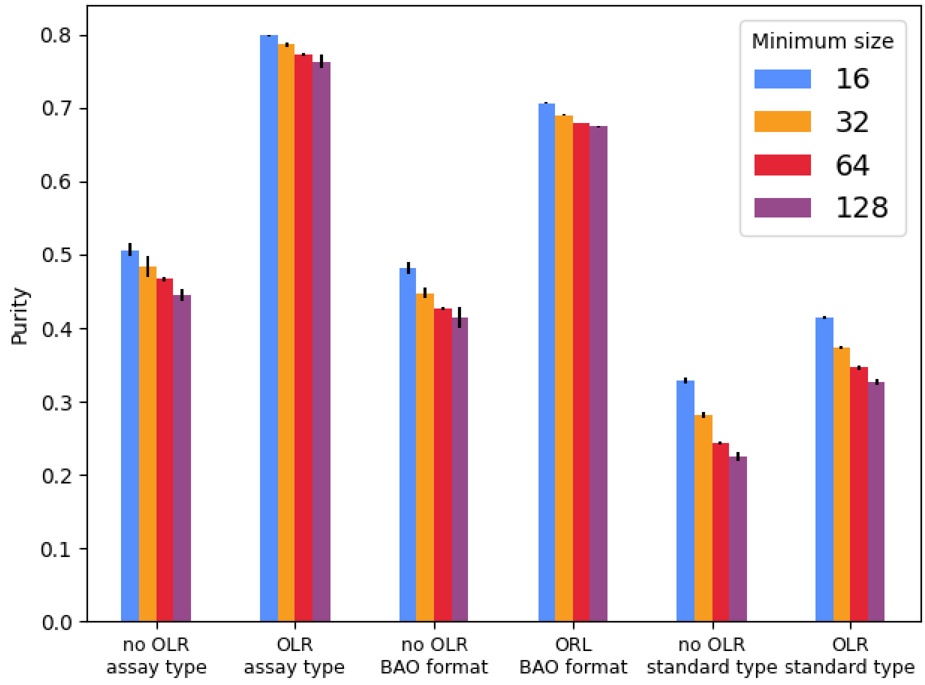

Figure SI 6, bar plot of the normalized purity for the labels assay type, BAO format, and standard type based on clustering with gte-Qwen2-1.5B-instruct embeddings. The purity is shown for models with and without outlier reduction (OLR) and with different minimum cluster sizes (16, 32, 64 and 128). Mean and standard deviation are based on independent clustering runs (n=3).
